# A Telemedicine Robot System for Assisted and Independent Living

**DOI:** 10.3390/s19040834

**Published:** 2019-02-18

**Authors:** Natasa Koceska, Saso Koceski, Pierluigi Beomonte Zobel, Vladimir Trajkovik, Nuno Garcia

**Affiliations:** 1 Faculty of Computer Science, University “Goce Delcev”, 2000 Shtip, Macedonia; saso.koceski@ugd.edu.mk; 2 DIIIE, University of L’Aquila, 67100 L’Aquila, Italy; pierluigi.zobel@univaq.it; 3 Faculty of Computer Science and Engineering, University “Ss Cyril and Methodius”, 1000 Skopje, Macedonia; trvlado@finki.ukim.mk; 4 Instituto de Telecomunicações, Universidade da Beira Interior, 6200-001 Covilhã, Portugal; ngarcia@di.ubi.pt

**Keywords:** assistive robotics, elderly care, mobile robot system, shared control, telepresence robot

## Abstract

The emerging demographic trends toward an aging population, demand new ways and solutions to improve the quality of elderly life. These include, prolonged independent living, improved health care, and reduced social isolation. Recent technological advances in the field of assistive robotics bring higher sophistication and various assistive abilities that can help in achieving these goals. In this paper, we present design and validation of a low-cost telepresence robot that can assist the elderly and their professional caregivers, in everyday activities. The developed robot structure and its control objectives were tested in, both, a simulation and experimental environment. On-field experiments were done in a private elderly care center involving elderly persons and caregivers as participants. The goal of the evaluation study was to test the software architecture and the robot capabilities for navigation, as well as the robot manipulator. Moreover, participants’ reactions toward a possible adoption of the developed robot system in everyday activities were assessed. The obtained results of the conducted evaluation study are also presented and discussed.

## 1. Introduction

Personal service robot, or service robot for personal use, is a robot used for a non-commercial task, usually by lay persons [1]. Examples of such robots are domestic servant robots, automated wheelchair, personal assistant robots, and pet exercising robots. Main categories of personal service robots include robots for domestic tasks (vacuum and floor cleaning, lawn-mowing, pool, window cleaning robots, etc.), entertainment robots (toy robots, educational robots, etc.), elderly and handicap assistance (robotized wheelchairs, assistive device, etc.), robots for transportation and robots for home security and surveillance. International Federation of Robotics (IFR) estimates that in the period of 2019–2021 sales of service robots for personal and domestic use will increase by a 31% CAGR (Compound Annual Growth Rate), on average, per year, most of which will be on robots for the elderly and handicap assistance (with a predicted sale of 34,400 units in the mentioned period) [2].

Robotic assistants used in the elderly and the disabled care has emerged as a major market of the future. The combination of low birth rates and higher life expectancies, in most countries, has resulted in more elderly people than, both, children and people capable of working, over a given period of time. By 2050, the number of people in the world who are aged over 60, is projected to grow more than double, from 605 million to 2 billion, representing 16% of the world’s population [3]. This will increase the health and societal costs, which can affect the economic growth in countries affected by this phenomenon.

The upcoming ageing society is demanding technological solutions that will help to overcome these issues. Assistive robotics for the elderly includes a range of services and tasks to assist the elderly, and the professional caregivers, in everyday activities. Many personal service robots currently available on the market are designed to perform specific tasks. Typical examples are the routine tasks, such as eating, drinking, manipulating objects, reminder for taking medications, maintaining a shopping list, as well as emergency notification, and navigation (PEARL [4], CompanionAble [5], CareBot [6], Kompai [7], Care-o-Bot [8], PR2 [9]). The main interaction modality used in these systems is a verbal communication using voice recognition software. Other robotic systems can collect vital signs data, and assist medical professionals in the exchange of information and enhanced service to elderly (InTouch Vita [10], CompanionAble [5], CareBot [6], and Care-o-Bot [8]). Assistive robots, the so-called rehabilitation robots can assist people with cognitive, sensory, or motor impairments (including robotic wheelchairs, walkers, and motor skill enhancement armatures) [11]. 

Elderly people do not only need a healthcare support, but also need a social support. Social isolation and loneliness are closely associated with negative physical, mental or emotional health of the elderly [12,13,14,15,16], which can lead to higher rates of dependency for health services. This could potentially place an additional burden on healthcare system in the countries experiencing a growth in the elderly population. In addition, many studies show that social interaction have a positive impact on the general mental and physical wellbeing, and decrease the occurrence of specific age-correlated diseases [17,18,19,20].

Some mobile robotic systems are designed to offer companionship to older people and support them via social interactions (CompanionAble [5], CareBot [6], TRIC [21], Kompai [7], VGo [22], Anybots [23], Giraff [24], and Care-o-Bot [8]). They are typically equipped with video camera, microphones, and display. These telepresence robots act as video-conferencing systems enabling communication with caregivers, relatives, and healthcare professionals. They enable a person to be virtually present at the elderly’s residence and to move around as if he/she was physically there. They are not intended to replace the human presence, but rather to extend it for purposes of social interaction. Such robot proactively engages users in a social manner, interact with them while giving assistance and support in certain activities of daily living and care, encourages them while performing some difficult tasks, or just greets the elderly when they meet. 

Despite some reservation amongst caregivers and families, regarding the use of these robot systems in the residences of the elderly, different studies have confirmed the positive attitude of the elderly towards having a robot in their vicinity [25,26,27,28]. Assistive robots could potentially enhance the health and psychological well-being of the elderly people and decrease the working load of healthcare professionals [29]. Our previous study, focused on the acceptance of the telepresence robot systems, also confirmed this finding [30]. However, the robots that are capable of enhancing more aspects of well-being, are more acceptable than the ones that cover only one [29]. 

Most of the afore-mentioned robots offer multiple services for the users (CompanionAble, CareBot, Kompai, Giraff, etc.). However, only two of them, the Care-o-Bot and the PR2, have manipulation skills—they are equipped with arms that are used for grasping and manipulating objects in various environments. This is considered to be a very important feature, because the capability of catching and carrying small objects is highly preferred by the elderly [31,32]. However, these robots with manipulation capabilities, are very expensive. For example, the configuration of the PR2 robot, with a single arm, costs about $285,000 and its double-arm variant costs about $400,000. In addition to the high cost, these robots have their own limitations, such as the absence of both social and healthcare capabilities, inability to pick-up objects from high shelves, as well as problems with thresholds, during indoor navigation.

Having in mind these limitations of the existing solutions, we have designed and developed a low-cost assistive telepresence robot system for facilitating the health care of the elderly, and improving the quality of life of the elderly and handicapped. The developed robot, along with its functionalities, permit various interactions in a remote environment, like navigation, fetch and carry small objects, measuring vital parameters of an elderly person, reminder, calendar, and interpersonal communication. The potential users of the robot system are not only the elderly but, also professional caregivers. The robot can be remotely controlled by a distant person (e.g., a professional caregiver), and can perform some activities as if he/she was physically present at the elderly’s residence. 

Section 2 describes the design of the assistive telepresence robot. The robot modeling and control are elaborated in Section 3. Section 4 presents the evaluation phase, where a software architecture and the robot’s capabilities for navigation, as well as the manipulator functionality, were evaluated by the elderly and professional caregivers. Results of simulation and real-world experiments are described in Section 5. Finally, conclusions are presented in Section 6.

## 2. Design of the Developed Robot

### 2.1. The Robot’s Structure 

**Figure 1:**
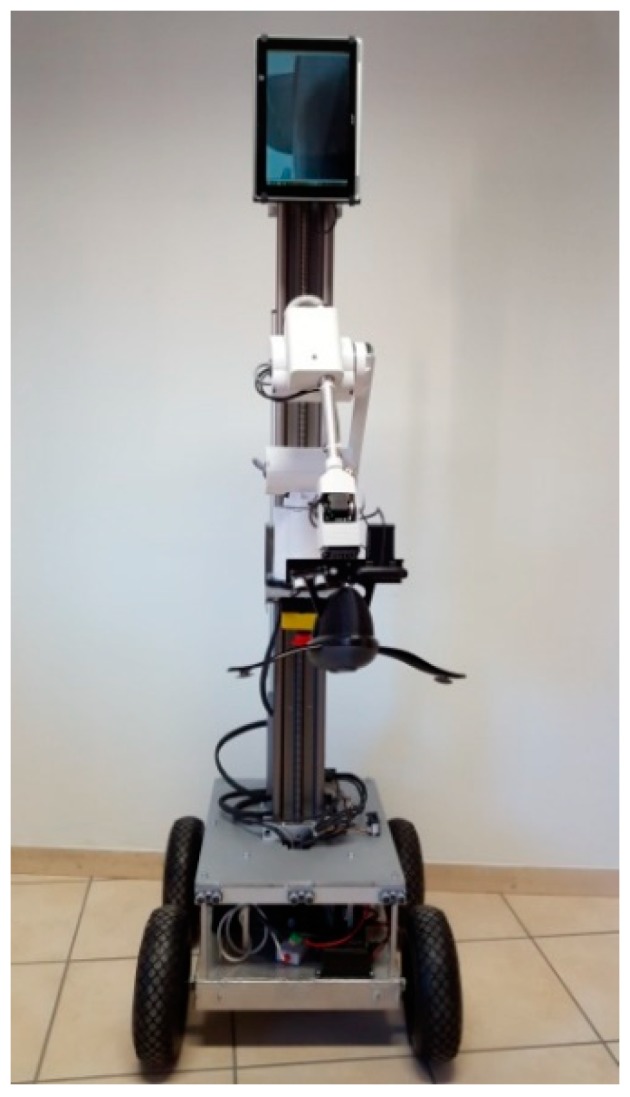
Developed assistive telepresence robot.

Figure 1 shows the developed telepresence robot, which is composed of a mobile robot base, a robot body, a robot arm, and a robot head.

The mobile base is driven by four pneumatic wheels, 10 inch in diameter. Two 12 V LiFePO_4_ (Li-ion) batteries with 20 Ah, aimed at powering the robot system, are placed inside the mobile base. In addition, an array of twelve ultrasound sensors and wide-angle cameras, are mounted on the mobile base, to facilitate obstacle avoidance and improve the visual feedback during navigation.

The robot base also bears two linear actuators, from IGUS manufacturer [33], which are vertically positioned, and mimic the robot’s body. Both actuators have a full length of 100 cm. The effective stroke of the actuators is limited to 80% of their full-length, due to safety reasons. The rest 20% of the stroke are defined as a dead zone. One of the actuators is used for the vertical positioning of the robot arm and the other for the positioning of the tablet. The position of the actuators is independently controlled via the integrated linear encoders, with a precision of 0.1 mm. The robot is equipped with a serial six-axis kinematic arm, based on the Mover arm from Common Place Robotics [34], with a custom modification of its end-effector. The arm weighs 3.5 kg, has a reach of 600 mm, and can lift objects up to 600 g. Considering the effective stroke of the actuator and the reach of the robot arm, the robot is capable of picking up objects from the floor, as well as picking up objects from high shelves, up to 210 cm. To enable a precise object grasping, an ultrasound sensor and a web camera are placed in proximity to the gripper. 

The 10’’ tablet acts as a robot head. The tablet can be used during interpersonal communication with family members, friends or caregivers, who can virtually visit the elderly and interact with them in their living environment. The tablet camera can also be used during navigation task, for providing visual feedback to the operator. In order to increase the comfortableness of verbal interaction with the robot and maintain eye contact, we have made the position of the robot head adjustable. It can be moved, vertically, using the linear actuator, and it can be positioned from 60 cm, up to 180 cm from the ground. Moreover, in order to increase the field–of-view during communication with users, the robot head can be paned (from −60° to +60°) and tilted (from −20° to +20°) by the means of two servo motors.

### 2.2. Control Software Architecture

Availability and affordability of myriad high quality sensors, capable of perceiving the environment from various aspects, facilitates increasing the level of autonomy, as well as improving the efficiency of today’s assistive and social robots. However, despite the increased level of autonomy, some complex tasks, such as door-crossing, sharp turns, or cognitively demanding tasks still require human guidance to guarantee safety and efficiency. This becomes even more important in application scenarios where the robot interacts with the elderly and disabled persons.

Depending on the level of the robot’s autonomy, human–robot interaction could range from teleoperation control, through a safeguarded and shared-control operation, till autonomous control [35]. Considering the safety requirements during the interaction with elderly and disabled persons, the developed robot can be operated in two modes—manual teleoperation, or by using shared control paradigm. In the first mode of working, the operator is the one who has the complete control over the robot, for the entire period of operation. In the second mode, the robot receives the high-level instructions from the operator, and performs the low-level tasks, autonomously. More specifically, the operator instructs the robot in two ways, (1) by giving a general direction of movement or by specifying a destination; and (2) by selecting one or more primitives, ordered in a sequence, from a set of pre-defined high-level primitives, such as, TakeMedicineBox, WallFollowing, DoorPasing, etc. 

However, even when the robot is performing a low-level task, the operator can interrupt its execution, at any moment, if necessary. The implemented control architecture follows the Model-View-Controller pattern and it enables a reactive control, i.e., closed-control loop and autonomous behavior and safety management, even when the communication channel between the operator and the robot is interrupted (due to a network failure, for example). 

Among other low-level modules, the developed control architecture, implements the modules for robot navigation. For this purpose, the robot was modeled and evaluated through simulations, first, and later on via real-world experiments.

## 3. Robot Modeling and Control

The developed robot implements a skid-steering mechanism, based on a velocity difference between the inner and the outer wheels. Each wheel is motorized, i.e., tightly coupled, with the individual traction motor bolted on to the robot chassis. Skid-steering offers flexibility in motion, from point turning to straight steering, without an explicit steering mechanism. Regardless of the control method, the robot wheels are not steered, therefore, lateral skidding or slippage could occur, and must be taken into account. The quantity of slippage and, consequently, the rate of the turn, depend mainly on the tire–ground interaction.

### 3.1. The Robot Model

#### 3.1.1. Kinematic Model

**Figure 2:**
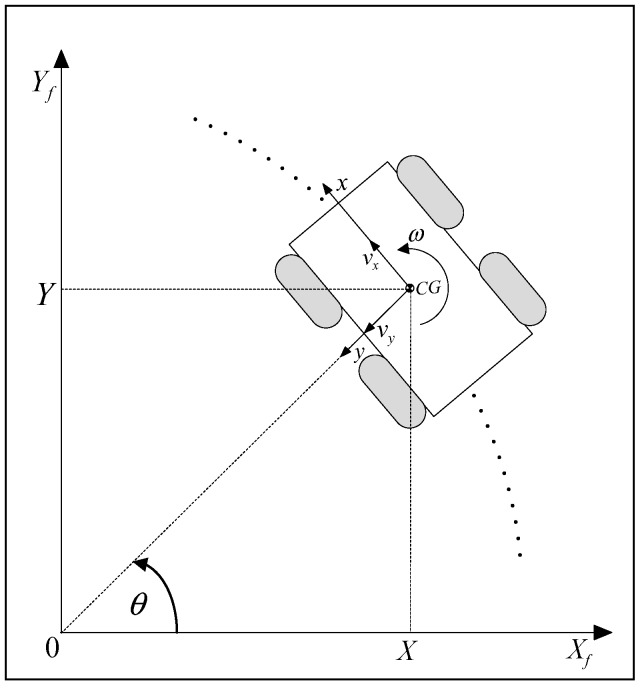
A skid-steered vehicle on a circular path.

Figure 2 shows a skid-steering vehicle moving on a circular path, about an instantaneous center of rotation (ICR), i.e., the origin of the fixed, frame. The local frame (robot frame) is attached to the vehicle’s center of gravity (CG), where x is the longitudinal axis, y is the lateral axis, $v_{x}$ and $v_{y}$ are the longitudinal and lateral velocities, respectively, ω is the angular velocity. The transformation of the local velocities to absolute velocities is as follows [36]:

(1) $$\left[\begin{array}{c} \dot{X}\\ \dot{Y} \end{array}\right]=\left[\begin{array}{cc} \cos\theta & -\sin\theta \\ \sin\theta & \cos\theta \end{array}\right]\left[\begin{array}{c} v_{x}\\ v_{y} \end{array}\right]$$

or

(2) $$\left[\begin{array}{c} \dot{X}\\ \dot{Y} \end{array}\right]=R(\theta )\left[\begin{array}{c} v_{x}\\ v_{y} \end{array}\right]$$

Further, this kinematic model can be augmented with the angular velocity $\dot{\theta }=\omega$ [37]. 

The generalized robot state vector describing the robot position X, Y, and orientation θ, with respect to the fixed frame, can be written as $q={\left[\begin{array}{ccc} X & Y & \theta \end{array}\right]}^{T}$. Assuming that the robot moves with a velocity vector $\dot{q}$, the robot motion in the local frame can be described with the vector $\eta =[\begin{array}{ccc} v_{x} & v_{y} & \omega \end{array}]$. From Figure 2 we can easily find the following mapping [38]:

(3) $$\eta =J(q)\dot{q}$$

where the matrix $J=\left[\begin{array}{cc} R^{T}(\theta ) & 0\\ 0 & 1 \end{array}\right]$. This kinematic model is described in terms of the longitudinal, lateral, and angular velocity, being commanded by the control system. Hence, it is suitable for planning and control. On the other hand, it does not take into account the skidding and slipping. The lateral sliding velocity $v_{y}$ can be relatively small and can be neglected ($v_{y}=0$). This fact has been used in Reference [39] for the development of an experimental kinematic model of a skid-steering robot.

#### 3.1.2. The Dynamic Model

**Figure 3:**
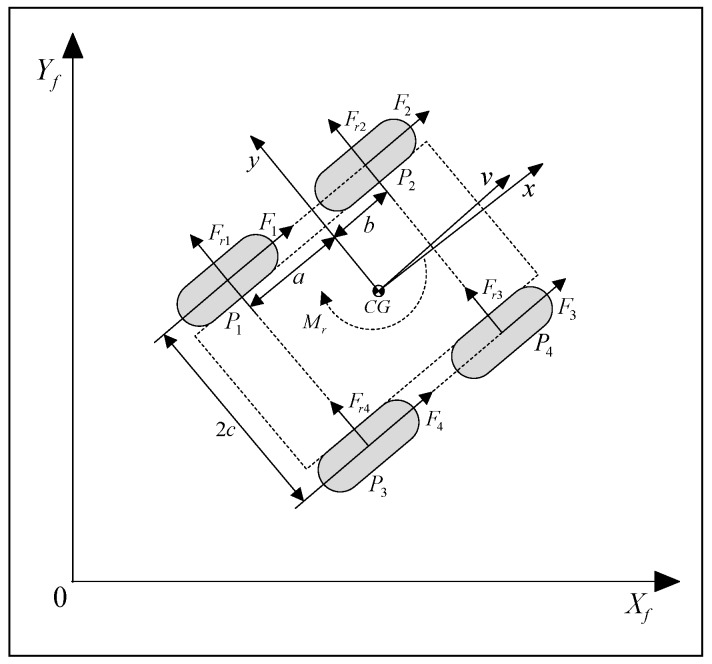
The active and reactive forces.

The dynamic model described in Reference [37] is given by

(4) $$M(q)\ddot{q}+F(\dot{q})=B(q)\tau$$

where M is a constant, positive definite inertia matrix, $F(\dot{q})$ is the vector of the resultant reactive forces and torque, B(q) is the input matrix, and $\tau =[\begin{array}{cc} \tau_{l} & \tau_{r}{]}^{T} \end{array}$ is the input vector with the torques produced by the left and right side pair of wheels, respectively. These torques produce the active forces $F_{i}$(i=1,2,3,4), see Figure 3.

The reactive lateral forces $F_{ri}$(i=1,2,3,4) are described using the Coulomb friction model:

(5) $$F_{ri}(v_{yi})=\mu_{i}N_{i}\;\mathrm{sgn}(v_{yi})$$

where $\mu_{i}$ and $v_{yi}$ determine friction and lateral velocity of the wheel–surface contact point $P_{i}$, $N_{i}$ is the wheel ground contact force resulting from gravity, and sgn is the sign function. 

For control purposes, it is suitable to rewrite the dynamic model from Equation (4), in terms of the vector η [38]:

(6) $$\bar{M}\dot{\eta }+\bar{C}\eta +\bar{F}=\bar{B}\tau$$

where

$$\bar{M}=\left[\begin{array}{ccc} m & 0 & 0\\ 0 & m & 0\\ 0 & 0 & I \end{array}\right],\;\;\bar{C}=\left[\begin{array}{ccc} 0 & -m & 0\\ m & 0 & 0\\ 0 & 0 & 0 \end{array}\right]\omega ,$$

$$\bar{F}={\left[\begin{array}{ccc} 0 & F_{r} & M_{r} \end{array}\right]}^{T},\;\;\bar{B}=\frac{1}{r}\left[\begin{array}{cc} 1 & 1\\ 0 & 0\\ -c & c \end{array}\right]$$

with

$$F_{r}=\sum_{i=1}^{4}F_{ri}(v_{yi}),\;M_{r}=-a\sum_{i=1,4}F_{ri}(v_{yi})+b\sum_{i=2,3}F_{ri}(v_{yi}),$$

and $r=r_{i}$(i=1,2,3,4), supposing that the radii of the wheels are the same.

### 3.2. Robot Control

**Figure 4:**
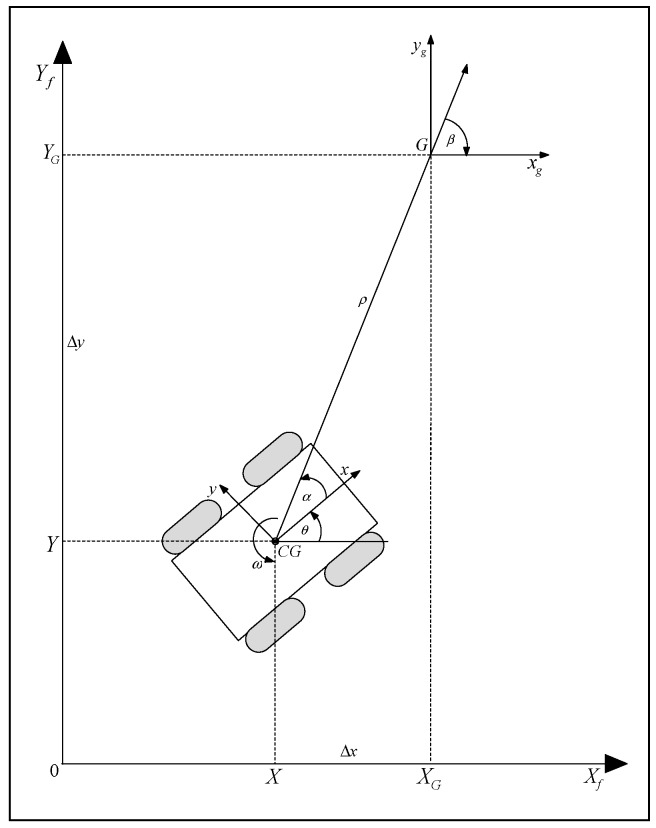
Kinematic control.

The implemented robot controller should enable path and motion planning. Namely, it should plan the optimal path, from the start till the end position, based on the a priori knowledge it has about the environment and the robot characteristics. It should also implement an accurate motion control along the path, which means a dynamic response to the changes in the environment [40]. The most common way to define the path of a mobile robot is by a series of waypoints that the robot needs to pass (Figure 4).

#### 3.2.1. Control Objective

The velocity control methods exhibit easiness and robustness. Rewriting the kinematic model in terms of the generalized robot vector q, gives:

(7) $$\left[\begin{array}{c} \dot{X}\\ \dot{Y}\\ \dot{\theta } \end{array}\right]=\left[\begin{array}{cc} \cos\theta & 0\\ \sin\theta & 0\\ 0 & 1 \end{array}\right]\left[\begin{array}{c} v\\ \omega \end{array}\right]$$

The geometry of the control problem is shown in Figure 4. The relative errors between the robot and the next waypoint (goal G), in polar coordinates, are as follows:

(8) $$\rho =\sqrt{\Delta x^{2}+\Delta y^{2}}$$

(9) α=−θ+atan2(Δy,Δx)

(10) β=−θ−α

This yields a system description in the new polar coordinates [41]:

(11) $$\left[\begin{array}{c} \dot{\rho }\\ \dot{\alpha }\\ \dot{\beta } \end{array}\right]=\left[\begin{array}{cc} -\cos\alpha & 0\\ \sin\alpha /\rho & -1\\ -\sin\alpha /\rho & 1 \end{array}\right]\left[\begin{array}{c} v\\ \omega \end{array}\right]$$

#### 3.2.2. Control Law

Design control signals v and ω, which drives the robot to the next waypoint, using the linear control law [41]:

(12) $$v=\kappa_{\rho }\rho$$

(13) $$\omega =\kappa_{\alpha }\alpha +\kappa_{\beta }\beta ,$$

The above equations along with Equation (11), generates a closed-loop system described by:

(14) $$\left[\begin{array}{c} \dot{\rho }\\ \dot{\alpha }\\ \dot{\beta } \end{array}\right]=\left[\begin{array}{c} -\kappa_{\rho }\rho \cos\alpha \\ \kappa_{\rho }\sin\alpha -\kappa_{\alpha }\alpha +\kappa_{\beta }\beta \\ -\kappa_{\rho }\sin\alpha \end{array}\right].$$

It is shown that the system is exponentially stable, if $\kappa_{\rho }>0$, $\kappa_{\beta }<0$ and $\kappa_{\alpha }-\kappa_{\rho }>0$ [41].

## 4. The Evaluation Study

The developed assistive telepresence robot was tested in a simulation and in an experimental environment. The aim of the performed evaluation study was multifold—to test the software architecture and the robot capabilities for navigation, to test the robot manipulator and to assess users’ reaction toward a possible adoption of the developed robot system in everyday activities.

### 4.1. Participants

Participants were recruited from a private elderly care center Nursing Home “Idila Terzieva” [42], which served as an experimental environment. In total, thirty-one participants were involved in the study—26 elderly people (14 male and 12 female, aged between 55–75 years; M = 64) and 5 professional caregivers who were employed in the studied nursing home (aged between 35–49 years; M = 40). All subjects gave their informed consent for inclusion before they participated in the study. None of the users had any previous experience in robot manipulation and control.

### 4.2. Procedures

First, the participants completed a study agreement form, describing the aim of the study, as well as their rights. Then, they completed a general demographics and technology experience questionnaire (regarding usage of smart phones, tablets, computers, etc.). A 5-point Likert scale (1 indicating “no experience”, and 5 indicating “daily experience”) was used for the technology experience questionnaire. The mean score was 2.2, indicating a moderate low-technology experience. 

Since, none of the users had any prior experience with a robot system, they had a training session before the experiment. The training consisted of a lecture explaining the robot’s working principles and the functions of the control software. Then, the participants had free practice sessions where they drove the robot in the laboratory environment. They were constantly monitored by the evaluator, who helped them, if some risk situations occurred.

Afterwards, the robot was placed in a real evaluation environment. This was in line with several research that addressed the importance of testing the robot platform in real environments [43,44].

Several experiments were conducted during the evaluation. The first experiment aimed at testing the software architecture and comparing the shared-control paradigm and the manual control. Participants were asked to operate the robot using both control modes (manual and shared-control).

The second experiment addressed the robot capabilities for navigation in an environment cluttered with obstacles, while operating in the shared-control mode. During the experiment, path length and its smoothness, as well as avoidance of collisions with the obstacles in the environment were observed. For the evaluation purposes, several environment configurations were created. The same configurations were used in both the simulation and the real-world experiments.

The third experiment focused on the manipulator function. In particular, the robot manipulator was used to fetch some small objects, like a box of medicines, a can, and a bottle of water.

At the end of the evaluation, semi-structured interviews were conducted, to assess the user reaction to the developed robot system, in general, and different aspects of robot interaction, related to the usefulness, ease of use, and acceptability of the system.

## 5. Simulation and Experimental Environment Results

### 5.1. First Experiment—Comparison of the Shared and Manual Control

**Figure 5:**
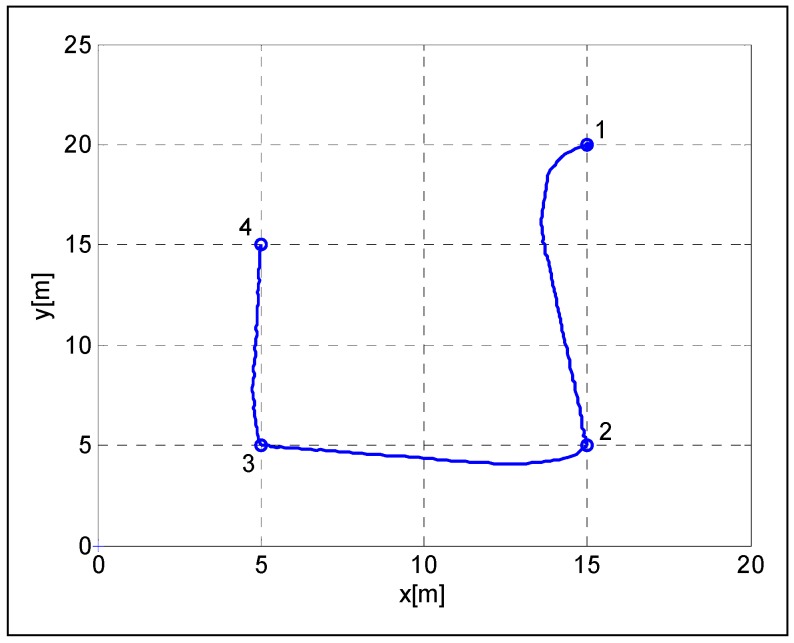
Robot trajectories in the way-point navigation task.

**Figure 6:**
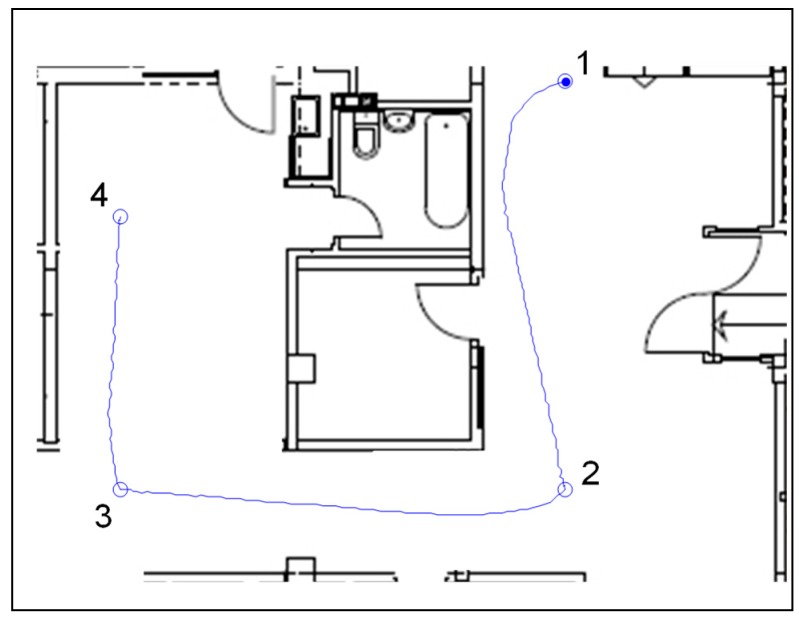
Robot trajectories on a ground plan.

In the simulation environment, the presented control algorithm was tested in a way-point navigation task. Four way-points 1(15m,20m), 2(15m,5m), 3(5m,5m), and 4(5m,15m) were chosen on an obstacle free path, in accordance with the ground plan, as shown in Figure 5 and Figure 6. The gains for the kinematic controller were chosen to be $\kappa_{\rho }=3$, $\kappa_{\alpha }=8$, and $\kappa_{\beta }=-1.5$ [37]. The robot speed was limited by $v_{\max}=1.5\;m/s.$ The robot’s initial heading was θ=0 degrees. As shown in Figure 5, the robot had successfully passed the way-points and finished the task. 

**Figure 7:**
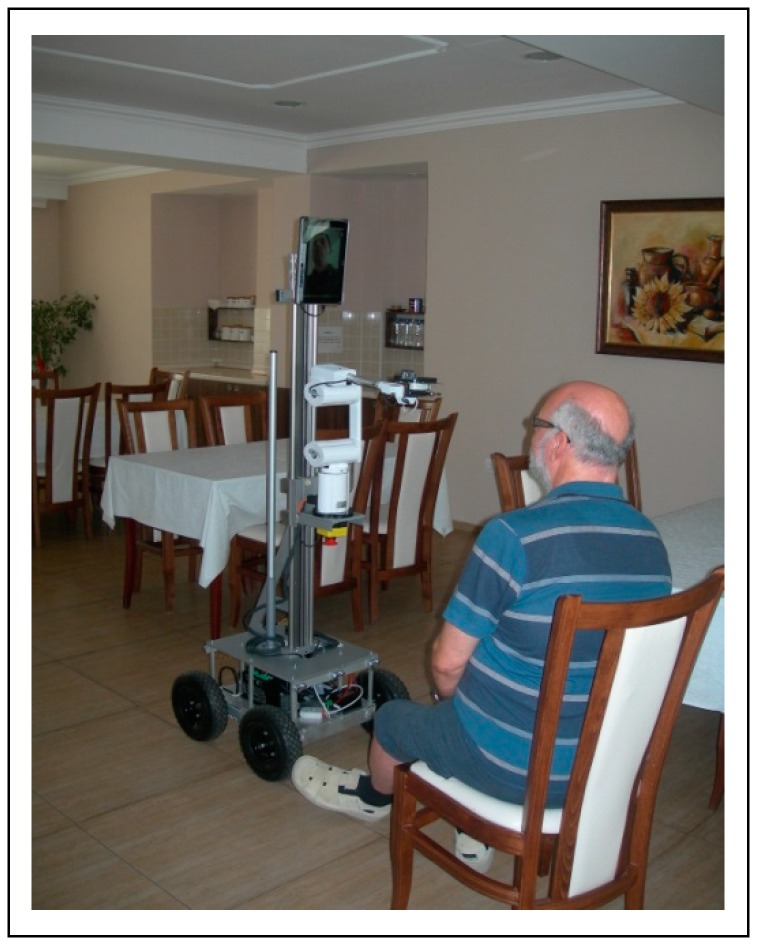
Experimental environment.

**Figure 8:**
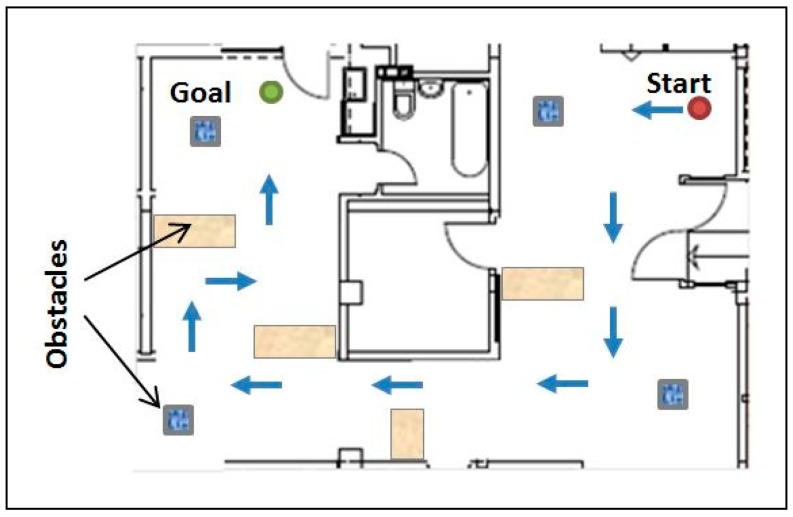
Evaluation environment for the navigation scenario.

Afterwards, the robot was placed in a real evaluation environment (Figure 7) and the participants were asked to move the robot from “Start” to “Finish”, avoiding the obstacles on the way. Intentionally placed carton boxes were used as obstacles (Figure 8). 

Each participant had conducted two runs, one using the manual and the other using the shared-control algorithm. During each run, collisions with the objects were counted, and times to finish the missions were recorded.

Moreover, the level of user engagement, during the shared-control mode of operation, was also measured. The level of engagement was simply defined as a percentage of the total task duration during which the user had complete control over the robot.

Average number of collisions and running times for both modes are presented in Table 1 and the results regarding the level of user engagement are presented in Table 2.

The presented results show that the implemented shared control mechanism helped the operators to significantly reduce the collisions. For the elderly, these varies from 7.85 (SD = 1.28) to 3.65 (SD = 0.98) (a two-sample *t*-test also showed a statistically significant difference between the two modes, *t* (26) = 6.88, *p* = 0.001), and for the caregivers it varied from 5.67 (SD = 1.13) to 2.13 (0.65) (a two-sample *t*-test also showed a statistically significant difference between the two modes, *t* (5) = 5.38, *p* = 0.001).

As a result of the collision reduction, the running time for each mission, was also reduced. The curvature of the followed trajectory was significantly lower during shared-control, for both groups of users.

### 5.2. Second Experiment—Static and Dynamic Obstacle Avoidance

One of the major concerns during robot navigation is obstacles detection and avoidance. For this purpose, ultrasound sensors—attached to the robot base—were used. A simulation environment created in Matlab, was also used in this experiment. The custom simulator was provided with an environment map with static obstacles that corresponded to the real environment. Moreover, the robot kinematics, as well as the sensors were modeled and implemented in Matlab.

**Figure 9:**
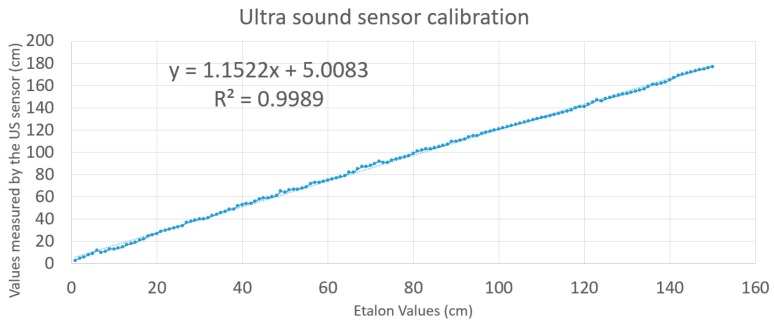
Ultrasound sensor calibration and model fitting.

A mathematical model was developed for the sensors used in the simulation, after the sensors were calibrated. In the calibration process the distances to a movable object, obtained with the firmware, were compared with the etalon (the ground truth). The etalon values were obtained using a Leica DISTO D2 200ft Laser Distance Measurer. Afterwards, a linear regression was performed and the result is shown in Figure 9.

The sensor model used in the simulation is expressed by the following equation:

(15) D=(k×d+n)+N

*D* is the distance given by the sensor, *d* is the real distance to the object (corresponds to the Euclidian distance), *k* = 1.1522 and *n* = 5.0083 are constants obtained by a linear regression, and *N* is the noise defined as:

(16) N=σ×G+m

where: *σ* = 1.3 cm is the standard deviation, *m* is the mean of the Gaussian law, and *G* is the Gaussian variable that can be computed by:

(17) $$G=\sqrt{-2\cdot \ln\left(R_{1}\right)}\cdot \cos\left(2\pi R_{2}\right)$$

where *R*_1_ and *R*_2_ are two variables, uniform in law, on [0,1].

**Figure 10:**
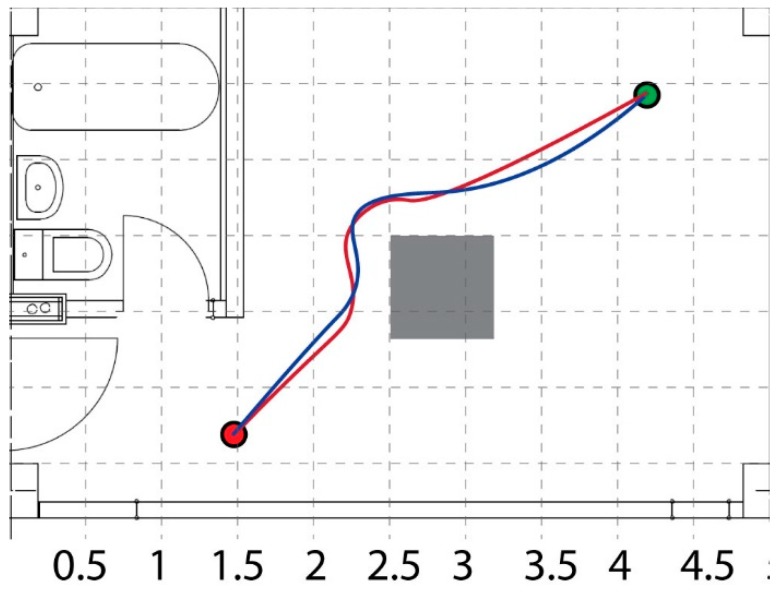
Obstacle avoidance.

The first test scenario, shown in Figure 10, aimed at presenting the robot capability to avoid obstacles on its way to the goal. Both, the paths obtained during simulation and the real-world experiment are depicted in the same figure (simulation path with red color and experimental path with blue color). The dimensions of the grid are expressed in meters.

**Figure 11:**
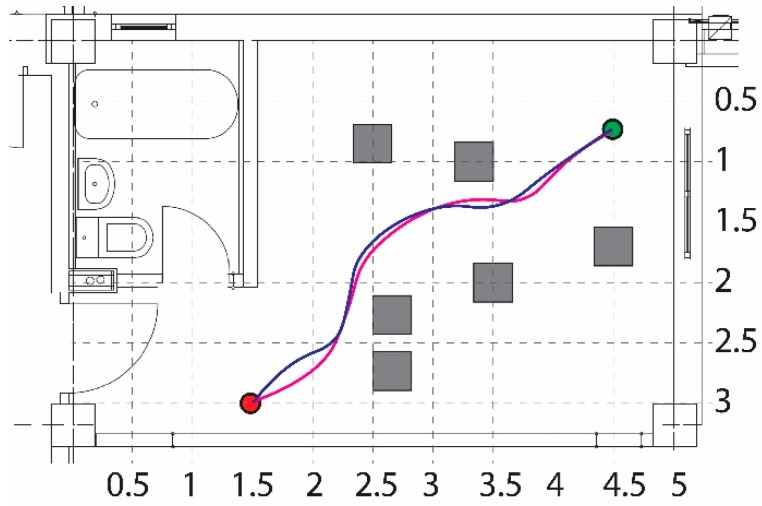
Obstacle avoidance for a cluttered environment.

The ability of the robot to reach the goal, by navigating in a cluttered environment, is presented in Figure 11. Both, the simulation and experimental paths are depicted in the same figure by the red and blue colors, respectively.

**Figure 12:**
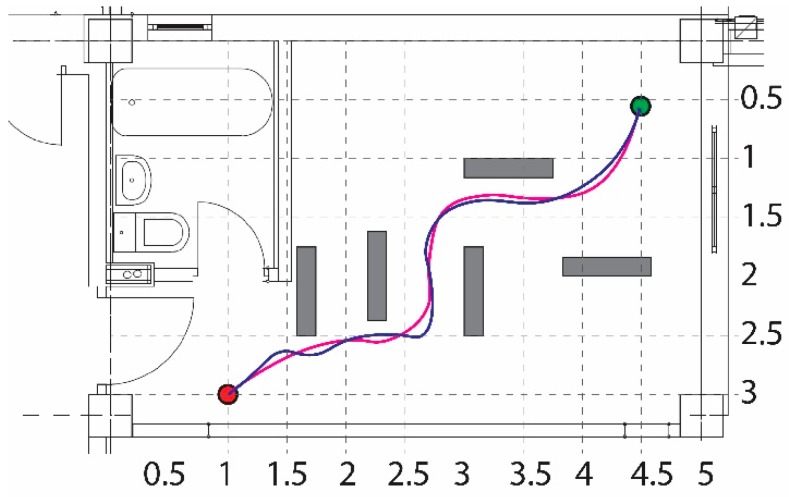
Obstacle avoidance in a corridor-like environment.

The next test scenario, shown in Figure 12, presents the case in which the robot was forced to reach the goal beginning at the start position, avoiding the obstacles (marked with grey), and by passing through a corridor-like passage. The simulated and experimental paths are marked by solid and dashed colors, respectively.

**Figure 13:**
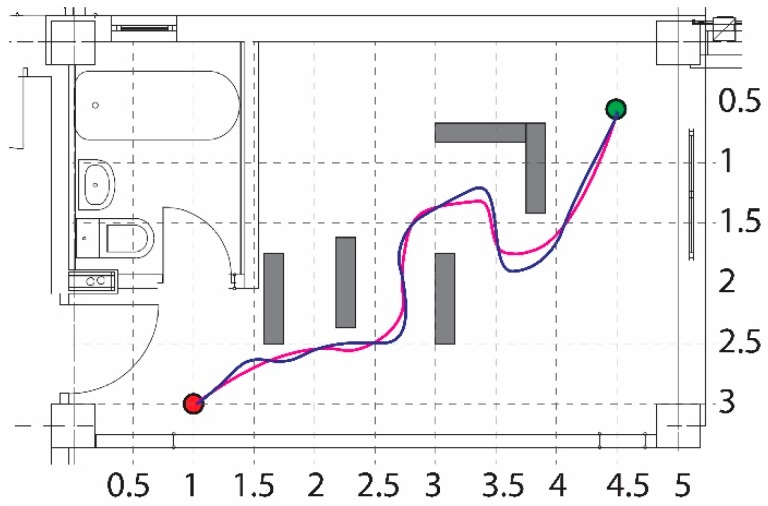
Corner avoidance.

Further on, we tested the robot’s ability to escape from corner-based dead-ways. This example is presented in Figure 13; the simulation path is drawn in red, and the experimental one in blue.

Lengths of the experimental and simulation parts in all evaluation scenarios are presented in the Table 3. As can be observed from the obtained results, the lengths of the paths obtained during the experimental scenarios in a real-world environment, had an almost equal length to the simulated ones. 

**Figure 14:**
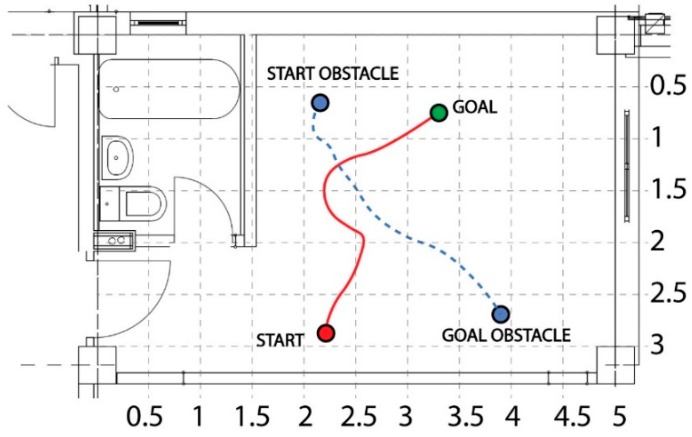
Dynamic obstacle avoidance.

**Figure 15:**
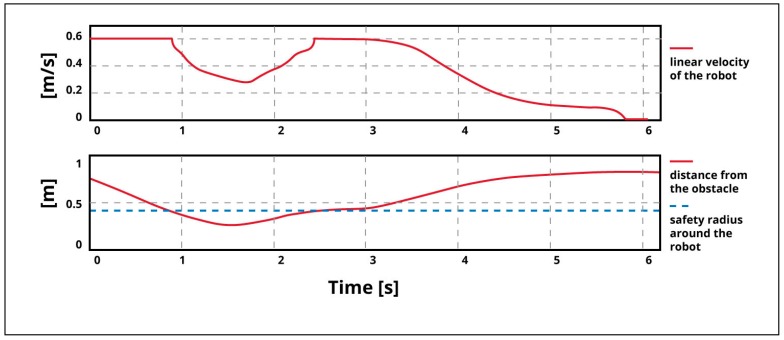
Linear velocity of the robot and its variations, depending on the distance separating the robot from the obstacle.

Afterwards, the control architecture and its ability to avoid dynamic obstacles was evaluated. Figure 14 shows the robot and the obstacle trajectories. At a low level, the robot implemented an adaptive neural fuzzy inference system (ANFIS) controller, in order to tune its velocity according to the position and distance from nearby obstacles. As can be seen from the figure, the robot avoided the dynamic obstacle by surrounding it from behind and attained its final target. The variation of the linear velocity of the robot and the distance separating it from the obstacle is presented in Figure 15. This graph shows that the robot started to decelerate when the distance to the obstacle was equal to the critical safety radius around the robot. After the obstacle was avoided, i.e., when the obstacle was out of the safety-zone around the robot, it accelerated again and started to decelerate by reaching the target.

### 5.3. The Third Experiment—Robot Manipulator Evaluation

The robot manipulator was tested on the task of picking up objects placed in three different places—a can was placed on a table, a medicine box was placed on the floor, and a bottle of water was placed on a high shelf. Ten users (5 elderly persons and 5 caregivers) participated in this evaluation. The robot was placed on the opposite side of the room where the objects were placed, and was instructed by the user, using a high-level command, to get positioned near the desired object. Then using a low-level command (e.g., TakeMedicineBox) the robot executed a grasp behavior. Ten trials were executed for each of the objects.

The fetching task was completed with a success rate of 88%. This rate is based on the robot being able to grasp the object, on the first attempt of each trial, while the failures include the robot being unable to grasp the object properly, due to inappropriate robot-positioning, inappropriate object-detection, or an error in robot-control.

### 5.4. Semi-Structured Interviews with the End Users

At the end of the evaluation, a semi-structured interviews, with all participants, were conducted. Our intention was to get user feedback on their interaction experience with the robot and to investigate their willingness to adopt the robot system.

General assessment of the robot system was positive. Most participants (73%) were willing to use the robot in their everyday life, as a tool for providing assistance and communication services. Majority of the caregivers (80%) reported that the robot could alleviate their workload. The elderly, also, did not have anything against a robot being in their vicinity, perceiving it only as an additional appliance in their surroundings.

The possibility of remote-monitoring of the elderly was preferred by 80% of the caregivers, emphasizing that visual communication would improve their surveillance, allowing them to monitor the physical condition of the elderly and their daily activities. Remote-monitoring and telepresence surveillance has always raised some questions about privacy issues. However, in our study the elderly people showed little concerns about this, as they live in a nursing home where they are constantly being monitored.

The manipulator functionality, was preferred by all participants. Regarding the ease-of-use, the participants evaluated the different aspects of their driving and communication experiences, on a scale of 1–5; “1” being “very difficult” to “5” being “very easy”. The average scores are shown in Table 4.

## 6. Conclusions

Design and validation of a low-cost assistive telepresence robot has been presented in this paper. The developed robot is intended for social interactions, assistance of elderly, and support of professional caregivers in providing better help and care. It offers multiple functionalities, like navigation, fetching and carrying small objects, measuring vital parameters of an elderly person, and providing reminders, a calendar, and interpersonal communication. 

The developed assistive telepresence robot was tested in a simulation and an experimental environment. The real-world experiments were performed in an elderly house with twenty-six elderly persons and five caregivers. The conducted evaluation study had multifold objectives—to test the software architecture and the robot capabilities for navigation, to test the robot manipulator, and to assess user reaction toward a possible adoption of the developed robot system in everyday activities.

The obtained results showed that the implemented shared-control paradigm significantly improved robot’s navigation capabilities and reduced obstacle collisions. Regarding the robot manipulator, the experiments showed that the robot was highly effective in capturing objects placed in different locations. 

A partial aim of the study was to investigate the acceptance of the robot developed and the effect of a direct experience with it. Overall, all participants showed positive attitudes towards the developed robot, and a willingness to use it in everyday life.

## Figures and Tables

**Table 1:** Comparison between the two modes of control—mean values and standard deviation.

Mode	No. of Collisions (St. Dev)		Running Time (s) (St. Dev)		Trajectory Curvature (m^−1^) (St. Dev)	
	Elderly	Caregivers	Elderly	Caregivers	Elderly	Caregivers
Manual	7.93 (1.45)	5.67 (1.13)	118.13 (17.35)	97.24 (12.18)	0.4 (0.23)	0.3 (0.16)
Shared control	3.65 (0.98)	2.13 (0.65)	104.35 (18.60)	85.17 (9.38)	0.21 (0.18)	0.12 (0.09)

**Table 2:** The level of user engagement during shared-control—mean values and standard deviation.

Mode	Level of Engagement Expressed as % of the Total Time Task (St. Dev)
**Elderly**	42 (9.19)
**Caregivers**	31 (5.18)

**Table 3:** Lengths of the paths during the experiments and the simulation.

Test No.	Path Length (meters)	
	Simulation	Experimental
1	3.84	3.86
2	3.98	4.02
3	5.41	5.95

**Table 4:** Results of the evaluation of the different aspects of the driving and communication experiences.

Issue	Evaluation (Average Score)	
	Elderly	Caregivers
General driving experience	3.6	4.4
Moving in a straight line	3.9	4.6
Turning	2.5	3.4
Door-passing	2.8	3.6
Communication experience	4.4	4.8
Visual feedback	3.8	4
